# Chemical Composition and Antibacterial Activity of the *Lippia origanoides* Kunth Essential Oil from the Carajás National Forest, Brazil

**DOI:** 10.1155/2021/9930336

**Published:** 2021-10-19

**Authors:** Fabiana Paiva Ribeiro, Mozaniel Santana de Oliveira, André de Oliveira Feitosa, Patricia Santana Barbosa Marinho, Andrey Moacir do Rosario Marinho, Eloisa Helena de Aguiar Andrade, Alcy Favacho Ribeiro

**Affiliations:** ^1^Faculty of Chemistry, Federal University of Pará, Ananindeua Campus, Tv. We Vinte e Seis, 2, Coqueiro, Ananindeua, Portugal 67130-660, Brazil; ^2^Adolpho Ducke Laboratory, Botany Coordination, Emílio Goeldi Museum, Av. Perimetral, 1901, Terra Firme, Belém, Portugal 66077-830, Brazil; ^3^Faculty of Chemistry, Federal University of Pará, R. Augusto Corrêa, 01-Guamá, Belém, Portugal 66075-110, Brazil

## Abstract

Species of the genus *Lippia* are rich in essential oils and have shown antibacterial properties, which may be related to oils' chemical composition. The present work aimed to evaluate the antimicrobial potential of *Lippia origanoides* Kunth against two bacteria strains: *Escherichia coli* and *Staphylococcus aureus*. Leaf essential oils were obtained by hydrodistillation in a modified Clevenger-type apparatus, and their chemical composition was determined by gas chromatography coupled to mass spectrometry (GC/MS) and flame ionization detection (GC/FID). We identified 28 compounds, representing 98.87% of the total concentration of the essential oil. The compounds identified at the highest concentrations were 1,8-cineole (35.04%), carvacrol (11.32%), p-cymene (8.53%), *α*-pinene (7.17%), and *γ*-terpinene (7.16%). The leaf essential oil of *L. origanoides* showed antibacterial action on biological isolates of *Escherichia coli* and *Staphylococcus aureus*. For *Escherichia coli*, the oil presented bactericidal action at concentrations of 5–20 *μ*L/mL. Regarding *Staphylococcus aureus*, the bactericidal effect was noted at 20 *μ*L/mL and the bacteriostatic action was noted around 2.5–10 *μ*L/mL. Given the results obtained, *L. origanoides* essential oil showed promising biological potential against Gram-positive (*Staphylococcus aureus*) and Gram-negative (*Escherichia coli*) bacteria, thus encouraging further studies on substances isolated from this species to contribute to the development of new antimicrobial drugs.

## 1. Introduction

The Verbenaceae family comprises approximately 34 genera and 1,200 species. The most representative genera are *Verbena*, *Lippia*, *Citharexylum*, *Stachytarpheta*, *Glandularia*, and *Duranta* [1, 2]. This family is widely distributed throughout the tropical and temperate zones of the Americas, Africa, and India. South America, Mexico, and the Andes specifically concentrate most of the diversity of species [3, 4].

Belonging to the Verbenaceae family, *Lippia origanoides* is native to some countries of Central America and northern South America, especially the Amazon region. It grows to approximately three meters in height and is rich in essential oils with potential medicinal properties [5–8]. Traditionally, medicinal and aromatic plants of the *Lippia* genus have shown several properties, such as analgesic, anti-inflammatory, antipyretic, sedative, antifungal, antihypertensive, larvicide, repellent, and antimicrobial activities. These plants have been used in the treatment of skin, gastrointestinal, and liver diseases [9–12].

The occasional development of microorganism resistance to commercially available drugs has encouraged studies on the antimicrobial potential of essential oils, which search for compounds that can prevent and treat diseases [13, 14]. Regarding bacteria, the effect of essential oils depends on some factors, such as the technique applied and the period of plant collection, which can influence the concentration of the compounds that will act on bacteria [15, 16].

The *Escherichia coli* bacterium belongs to the family of Enterobacteriaceae. It is a Gram‐negative rod-shaped bacterium, nonsporulating, nonmotile, or motile by peritrichous flagella. Its ideal growth temperature is 37°C. *E. coli* is transmitted through contaminated food, as a result of inadequate handling and hygiene practices [17]. It causes a variety of diseases, such as diarrhea, which is a major cause of infant mortality [18]. Recently, this bacterium has been developing resistance through genetic mutations, hence hindering its control [19, 20].

*Staphylococcus aureus* is part of the human microbiota [21] but can cause local diseases, such as skin infection, metastatic abscess formation, sepsis, peritonitis, and pneumonia [22]. Due to its adaptability and resistance, *S. aureus* has become one of the most important species in hospital and community-acquired infections [23, 24]. In this context, considering the Amazon biodiversity and the need to promote its sustainable use, the present work aimed to evaluate the chemical composition and *in vitro* antimicrobial potential of *L. origanoides* Kunth essential oil against *E. coli* and *S. aureus*.

## 2. Materials and Methods

### 2.1. Raw Material Collection

The collection of the botanical material *L. origanoides* Kunth was carried out during the flowering period at the Carajás National Forest, an environmental protection area (geographic coordinates: 05°52′, 06°33′S; 49°53, 50°45′W). Samples were provided by Chico Mendes Institute for Biodiversity Conservation (ICMBio) (collection authorization number 24852-1). Specimens of *L*. *origanoides* Kunth were collected using botanical techniques and deposited in the João Murça Pires Herbarium of the Museu Paraense Emílio Goeldi, in Belém (Pará, Brazil), under the registration number MG 201029.

### 2.2. Essential Oil Isolation

To isolate the *L. origanoides* essential oil, we used 20 g of samples in a modified Clevenger-type apparatus coupled to a refrigeration system that maintained condensed water at 12°C. The essential oil obtained was centrifuged, and the residual moisture was removed with anhydrous sodium sulfate. Samples were stored in amber glass ampoules in the absence of oxygen and kept in a refrigerated room at −5°C. The oil yield was calculated by relating the volume of oil obtained and the material mass used in the extraction process on a dry basis [25, 26].

### 2.3. Chemical Composition Analysis

Chemical compositions were evaluated using a gas chromatograph (GC) coupled to a DSQ-II single quadrupole mass spectrometer (MS) (Thermo Fisher Scientific, Waltham, Massachusetts, USA) equipped with a silica capillary column DB-5MS (30 m × 0.25 mm × 0.25 *µ*m) (Agilent Technologies, Santa Clara, CA, United States). The evaluation conditions were the following: the temperature increased from 60 to 240°C at 3°C/min; the injector temperature was 240°C; helium was the carrier gas (linear velocity of 32 cm/s, measured at 100°C); a 2 : 1000 aqueous solution of *n*-hexane was injected (0.1 *µ*L); and the temperature of the ion source and other parts was 200°C. The quadrupole filter was scanned in the range of 39–500 Da per second. Ionization was achieved using the electronic impact technique at 70 eV. The retention index of all volatile compounds was calculated using a homologous series of n-alkanes (C_8_–C_40_) (Sigma-Aldrich, San Luis, AZ, USA) according to Van Den Dool and Dec Kratz [27]. The components were identified by comparison of (i) the experimental mass spectra with those existing in reference libraries and (ii) their retention indices with those found in the literature [28, 29]. Volatile components were quantified by peak area normalization using FOCUS GC/FID, which was operated under the same conditions as GC/MS, except for the carrier gas, which was nitrogen, as previously reported by our research group [30].

### 2.4. Analysis of In Vitro Antimicrobial Activity

The antimicrobial activity of *L. origanoides* oil was evaluated by the microdilution method, as described by Pinheiro et al. [31, 32]. Tests were performed at the Bioassay and Microorganism Chemistry Laboratory (LaBQuiM) of the Federal University of Pará, using strains provided by the Evandro Chagas Institute (IEC). Two bacteria were used: *Escherichia coli* (ATCC 25922) and *Staphylococcus aureus* (ATCC 25923). For 1 liter of broth, 37 g of brain heart infusion (BHI) agar was used. Then, 5 g of bacteriological agar was added to 100 mL of BHI broth. After these dilutions, all media were autoclaved at 121°C for 15 minutes to ensure complete sterility and dispensed onto dishes while still warm, before solidification. In the preparation of the antibiotic control, 1 mg of ampicillin was dissolved in 1 mL of distilled water. Then, 5 *µ*L of this solution was diluted in 995 *µ*L of BHI broth in an Eppendorf tube. A concentration of 5 *µ*L/mL was thus obtained.

#### 2.4.1. Bacteria Activation

The bacteria tested in the assays were activated in a 9 cm diameter Petri dish containing BHI agar for 24 h. After this period, approximately three colonies of each bacterium were transferred, with the aid of a sterile swab stick, to a test tube containing 3 mL of BHI broth, and then incubated for another 24 h. Their concentrations were standardized to obtain a culture medium with approximately 1.0 × 10^4^ CFU/mL [33].

#### 2.4.2. Standardization of the Culture Media

A barium sulfate suspension was obtained by mixing solutions of H_2_SO_4_ 1% (9.95 mL) and BaCl_2_ 1% (0.05 mL). Then, the turbidity of the test tube containing the bacteria was compared with the barium sulfate standard. After this standardization, the tube presented a concentration of approximately 1.0 × 10^4^ CFU/mL [33].

#### 2.4.3. Sample Preparation

For sample preparation, 20 *µ*L of *L. origanoides* essential oil was dissolved in 80 *µ*L of dimethyl sulfoxide (DMSO). Then, 900 *µ*L of sterilized and properly homogenized BHI broth was added to this solution.

#### 2.4.4. Determination of the Minimum Inhibitory Concentrations (MICs)

Minimum inhibitory concentrations (MICs) were determined by the microdilution method, using 96-well plates arranged in twelve columns (1–12) and eight rows (A-H), with 100 *µ*L of BHI broth added to each well. Then, 100 *µ*L of the essential oil solution was poured into the first well of each column and homogenized. After that, successive dilutions were performed, removing 100 *µ*L from the first well and transferring this volume to the next well, always homogenizing the final solution. This procedure was repeated until the penultimate well of each plate was filled, from which 100 *µ*L was removed and discarded. The last row was used as control of the medium, and no essential oil was added to this solution. Finally, 5 *µ*L of the bacterial suspension was poured into each well, and the plates were incubated at 37°C for 24 hours. Assays were performed in triplicate, and the results (concentrations) were expressed in *µ*L/mL.

After the incubation time, the presence of microbial growth in the plates was verified by the presence of turbidity (red), so clear wells corresponded to no microbial growth. Results were verified with the aid of TTC dye (2,3,5-triphenyltetrazolium chloride). Plates without red coloration were re-inoculated and incubated at 37°C for 24 hours [34].

#### 2.4.5. Determination of the Minimum Bactericidal Concentrations (MBCs)

After checking MIC, we verified the type of activity presented in each concentration of *L. origanoides* oil (bacteriostatic or bactericidal). The determination of the minimum bactericidal concentration (MBC) was performed by inoculation of Petri dishes containing BHI agar. Then, they were incubated at 37°C for 24 hours [35–37].

## 3. Results and Discussion

### 3.1. Yield and Chemical Composition of the Essential Oil

**Figure 1:**
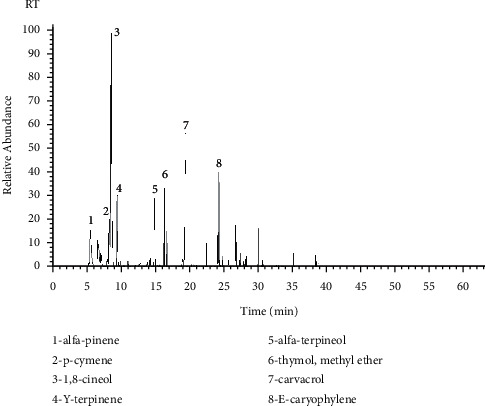
Ion-chromatogram (GC/MS) of the *L. origanoides* essential oil.

Approximately 0.6 mL of leaf essential oil was obtained, corresponding to an yield of 3%, which was higher than that obtained by Mar et al. [8]. The yield of *L. origanoides* may vary from 1 to 4.4% according to its geographical origin, extraction technique used, seasonal period, and rainfall rates [38–40].  Figure 1 shows the ion-chromatogram relative to the compounds identified in the essential oil of *L. origanoides*.

The chemical composition of *L. origanoides* essential oil is shown in Table 1. Oxygenated monoterpenes and monoterpene hydrocarbons were the major substances, which represented 56.57 and 35.73% of the compounds identified in this study, respectively. This result was similar to that observed by Mar et al. [8], in which the predominant class was also oxygenated monoterpenes (65%). Andrade et al. [41] also identified monoterpenes as the class with the highest concentration (90.3%) in the essential oil of *L. origanoides* collected in Minas Gerais (Brazil).

Ribeiro et al. [40] analyzed the chemical compositions of *L. origanoides* collected at different seasons of the year and found that the compound classes present in its essential oil may vary. For instance, monoterpene hydrocarbons, oxygenated monoterpenes, sesquiterpene hydrocarbons, oxygenated sesquiterpenes, and phenylpropanoids (cinnamates) may be present in the ranges of 9.4–46.5%, 13.5–62.2%, 17.5–31.0%, 3.3–52.8%, and 0.1–28.8%, respectively.

In the present study, we identified 28 compounds, whereas Stashenko et al. [38] found 139 substances in oils and extracts of *L. origanoides.* The major compounds found were 1,8-cineole (35.04%), carvacrol (11.32%), p-cymene (8.53%), *α*-pinene (7.17%), and *β*-terpinene (7.16%). Similarly, Tozin et al. [42], in samples of *L. origanoides* collected during the flowering period in the state of São Paulo (Brazil), identified 1,8-cineole as the main component. Also, in the work published by Da Silva et al. [43], the main substances found were 1,8-cineole (64.1%) and *α*-terpineol (12.0%), a result similar to that obtained by da Silva et al. [44].

In contrast, the chemical composition found in the present study was different from that of other publications. Carvacrol, for instance, was the major component found in *L. origanoides* collected in the city of Jardinópolis (São Paulo, Brazil), with a concentration of 26.28% [45]. In the essential oil of *L. origanoides* collected at Embrapa Western Amazon in Manaus (Amazonas, Brazil), the major compound was thymol (76.6%), whereas in *L. origanoides* collected in the city of Oriximiná (Pará, Brazil), the major compounds were carvacrol (38.6%) and thymol (18.5%) [46].

According to Rojas et al. [47], compound concentrations in the essential oils of *L. origanoides* may vary according to the collection period. For example, in June (rainy season), thymol and carvacrol had concentrations of 61.9 and 7.9%, respectively, whereas in February (dry season), their concentrations were approximately 44.7% and 16.8%, respectively. Also, Santos et al. [5] identified the following compounds at the highest concentrations in *L. origanoides* collected in the state of Piauí (Brazil): carvacrol (33.5–42.9%), *γ*-terpinene (8.0–10.5%), thymol (5.1–8.4%), methyl thymol (6.1–8.7%), and *p-*cymene (11.9–15.8%).

### 3.2. Antimicrobial Activity

The greatest antimicrobial activity was observed at the lowest values of MIC. Other authors also reported this behavior: MIC values ≤ 100 *µ*g/mL indicate strong antimicrobial activity [48, 49]. Our best result for antimicrobial activity was 2.5 *µ*L/mL against *S. aureus* strain, while the weakest was 5 *µ*L/mL against *E. coli*. Several studies [5, 46, 50] have shown that the essential oil of *L. origanoides* presents activity against the microorganisms *C. albicans*, *C. parapsilosis*, *C. guilliermondii*, *C. neoformans*, *T. rubrum*, *S. aureus* (MRSA BMB9393), *S. aureus*, *E. coli*, *L. casei*, *S. mutans*, *S. typhimurium*, *P. aeruginosa*, *B. cereus*, and *B. subtilis*. However, they report that these activities may be related only to the presence of carvacrol and thymol.

The bacteria tested showed variable susceptibility to the different concentrations of essential oil (Table 2). *L. origanoides* showed bacteriostatic action in the concentration range of 2.5–10 *μ*L/mL and bactericidal action against *S. aureus* beginning at 20 *μ*L/mL. Regarding *E. coli*, the essential oil showed bactericidal action at concentrations starting from 5 *μ*L/mL. Therefore, the MBC was 20 *µ*L/mL for *S. aureus* and 5 *µ*L/mL for *E. coli.* Studies on the chemical composition of *L. origanoides* essential oils have shown a great variety of components, such as thymol, *β*-caryophyllene, p-cymene (*E*)-nerolidol, trans-*α*-bergamotene, *α*-alaskene, *α*-pinene, *α*-humulene, caryophyllene oxide, and linalool [51]. This variety may be related to biological properties of the oil [52, 53], such as antimicrobial activity [54, 55].

According to Barreto et al. [56], *L. origanoides* essential oil in association with aminoglycosides may present a synergistic effect and be an appropriate alternative for antibiotic chemotherapy against diseases caused by methicillin-resistant *Staphylococcus aureus* (MRSA). Similarly, in a study on the potential antimicrobial effect of *L. origanoides* essential oil rich in thymol (76.6%) and ortho-cymene (6.3%) against *Aeromonas hydrophila,* Majolo et al. [54] obtained a MIC of 2500 *μ*g/mL and a MBC of 2500 *μ*g/mL.

Finally, the results obtained in this study indicated that one of the compounds responsible for the antimicrobial activity of *L. origanoides* essential oil may be 1,8-cineole, since several scientific publications have reported the antimicrobial properties of this substance [57–61]. For instance, Hendry et al. [57] obtained the following results using 1,8-cineole: MIC values of 16 *µ*g/L (suspension) and 512 *µ*g/L (biofilm) and MBC values of 256 *µ*g/L (suspension) and >512 *µ*g/L (biofilm) for *S. aureus*; MIC values of 64 *µ*g/L (suspension) and 128 *µ*g/L (biofilm) and MBC values of 64 *µ*g/L (suspension) and 256 *µ*g/L (biofilm) for *E. coli*.

Other compounds have had their antimicrobial activity reported. Studies have demonstrated that *α*-pinene also has antimicrobial activity against *S. aureus* and *E. coli* [62–64]. De Sousa et al. [65], for instance, obtained a MIC of 1.25 *μ*l/mL and 2.5 *μ*l/mL for *α*-pinene against *S. aureus* and *E. coli*, respectively. p-Cymene, another compound identified in the essential oil of *L. origanoides*, also has antimicrobial activity [66]. Namiecińska et al. [67] studied the antimicrobial effects of p-cymene on different strains. They obtained a MIC of 1000 *μ*g/mL for *S. aureus* ATCC 29213, 62.5 *μ*g/mL for *S. epidermidis* ATCC 12228, and 500 *μ*g/mL for *E. faecalis* ATCC 29212. *γ*-Terpinene, in the work by Krist et al. [68], showed good antimicrobial activity, since the germ count decreased by 40%. In addition, carvacrol acted against several strains, such as *E. coli, P. fluorescens, S. aureus, L. plantarum, B. subtilis, S. cerevisiae,* and fungus *B. cinerea* [69]. Guarda et al. [70] studied the antimicrobial potential of carvacrol and obtained MIC values of 225, 225, 375, 75, and 225 *μ*g/mL for *S. aureus*, *L. innocua*, *E. coli, S. cerevisiae,* and *A. niger,* respectively.

## 4. Conclusion

The chemical composition of the essential oil of *L. origanoides* Kunth collected during the flowering period proved to be rich in 1,8-cineole, indicating a possible correlation between the collection period and the biosynthesis of such a compound. We also observed a potential antimicrobial activity against Gram-positive and Gram-negative bacteria, suggesting a possible association of this behavior with the concentration of 1,8-cineole. Given the results, the essential oil of *L. origanoides* Kunth showed promising biological potential against pathogenic bacteria, thus encouraging further studies on substances isolated from this species to contribute to the development of new antimicrobial drugs.

## Figures and Tables

**Table 1:** Volatile constituents identified in *L. origanoides* Kunth essential oil.

RT	RI_*C*_	RI_*L*_	Constituents	Molecular formula	(%)
5.60	925	924	*α*-Thujene	C_10_ H_16_	0.49
5.84	932	932	*α*-Pinene	C_10_ H_16_	7.17
6.89	971	969	Sabinene	C_10_ H_16_	3.69
7.03	976	974	*ß*-Pinene	C_10_ H_16_	3.74
7.31	987	988	Myrcene	C_10_ H_16_	3.73
8.35	1017	1014	*α*-Terpinene	C_10_ H_16_	1.22
8.56	1024	1020	*p-*Cymene	C_10_ H_14_	8.53
8.76	1032	1026	1,8-Cineole	C_10_H_18_O	35.04
9.79	1057	1054	*γ*-Terpinene	C_10_ H_16_	7.16
15.23	1197	1186	*α*-Terpineol	C_10_H_18_O	4.36
17.20	1228	1232	Thymol, methyl ether	C_11_H_16_O	5.58
17.55	1242	1239	Carvone	C_10_ H_14_O	0.12
20.15	1298	1298	Carvacrol	C_10_ H_14_O	11.32
20.20	1321	1289	*p-*Cymen-7-ol	C_10_ H_14_O	0.10
22.42	1356	1349	Thymol acetate	C_12_H_16_O_2_	0.05
23.51	1373	1374	*α*-Copaene	C_15_H_24_	0.80
25.10	1413	1410	*α*-Cedrene	C_15_H_24_	0.07
25.38	1416	1417	(*E*)-Caryophyllene	C_15_H_24_	3.15
26.09	1420	1434	*γ*-Elemene	C_15_H_24_	0.25
26.12	1430	1432	*α*-trans-Bergamotene	C_15_H_24_	0.36
26.84	1452	1452	*α*-Humulene	C_15_H_24_	0.23
27.98	1478	1479	ar-Curcumene	C_15_H_22_	0.18
28.60	1492	1493	*α*-Zingiberene	C_15_H_24_	0.46
28.86	1495	1500	*α*-Muurolene	C_15_H_24_	0.10
29.04	1505	1505	*ß*-Bisabolene	C_15_H_24_	0.14
29.42	1507	1514	(*Z)*-*γ*-Bisabolene	C_15_H_24_	0.07
19.80	1515	1522	*σ*-Cadinene	C_15_H_24_	0.52
32.18	1578	1582	Caryophyllene oxide	C_15_H_24_O	0.24
Hydrocarbon monoterpenes					35.7
Oxygenated monoterpenes					56.5
Hydrocarbon sesquiterpenes					6.33
Oxygenated sesquiterpenes					0.24
Total					98.87

RT: retention time; RI_C_: retention index (on DB-5MS column); RI_L_: retention index from literature (Adams [28]).

**Table 2:** Result of the antimicrobial assays against *Staphylococcus aureus* and *Escherichia coli*.

Concentrations (*µ*L/mL)	*Staphylococcus aureus*	*Escherichia coli*
20.00	=	=
10.00	−	=
5.00	−	=
2.50	−	+
1.25	+	+
0.62	+	+
0.31	+	+
Control	+	+

+, no activity; −, bacteriostatic; =, bactericidal.

## Data Availability

The data sets used and/or analyzed during the current study are available from the corresponding author and will be delivered to responsible bodies on reasonable request.

## Supplementary Materials

Supplementary Materials This section contains data regarding collection and experiments.
