# Supplementary material for: Chemical Composition and Antibacterial Activity of the Lippia origanoides Kunth Essential Oil from the Carajás National Forest, Brazil
Source: Evid Based Complement Alternat Med. 2021 Oct 19;2021:9930336. doi: 10.1155/2021/9930336 (PMC8548111; doi:10.1155/2021/9930336)

**Supplementary material S1**

**Chemical Composition and Antibacterial Activity of the *Lippia origanoides* Kunth Essential Oil from Carajás National Forest-Brazil**

Fabiana Paiva Ribeiro^1^, Mozaniel Santana de Oliveira^2*^, André de Oliveira Feitosa^3^, Patricia Santana Barbosa Marinho^3^, Andrey Moacir do Rosario Marinho^3^, Eloisa Helena de Aguiar Andrade^2,3^ and Alcy Favacho Ribeiro^1^.

^1^Faculty of Chemistry – Federal University of Pará - Ananindeua Campus, Tv. We Vinte e Seis, 2 - Coqueiro, Ananindeua, 67130-660, PA, Brazil.

^2^Adolpho Ducke Laboratory - Botany Coordination, Emílio Goeldi Museum, Av. Perimetral, 1901, Terra Firme, Belém 66077-830, PA, Brazil

^3^ Faculty of Chemistry – Federal University of Pará, R. Augusto Corrêa, 01 - Guamá, Belém, 66075-110, PA, Brazil.

*Correspondence: mozaniel.oliveira@yahoo.com.br; Tel.: +55-91-988-647-823.

**ABSTRACT**

Species of the genus *Lippia* are rich in essential oils and have shown antibacterial properties, which may be related to their chemical composition. In this sense, the present work aimed to evaluate the antimicrobial potential of *Lippia origanoides* Kunth against two bacteria strains: *Escherichia coli* and *Staphylococcus aureus*. Leaf essential oils were obtained by hydrodistillation in a modified Clevenger-type apparatus, and their chemical composition was determined by gas chromatography coupled with mass spectrometry (GC/MS) and flame ionization detection (GC/FID). In general, 28 compounds were identified, representing 98.87% of the total concentration in essential oil, the compounds identified at the highest concentrations were: 1,8-cineole (35.04%), carvacrol (11.32%), p-cymene (8.53%), α-pinene (7.17%), and γ-terpinene (7.16%). The leaf essential oil of *L. origanoides* showed antibacterial action on biological isolates of *Escherichia coli* and *Staphylococcus aureus*. For *Escherichia coli*, the oil presented bactericidal action at concentrations of 5 - 20 μL/mL. Regarding *Staphylococcus aureus*, the bactericidal effect was noted at 20 μL/mL and the bacteriostatic action around 2.5 - 10 μL/mL. Given the results obtained, *L. origanoides* essential oil showed promising biological potential against Gram-positive (*Staphylococcus aureus*) and Gram-negative (*Escherichia coli*) bacteria, thus encouraging further studies on substances isolated from this species, in order to contribute to the development of new antimicrobial drugs.

**Keywords**: Natural products; Bioactive compounds; Oxygenated monoterpenes; Biological activity.

Collection and experiments

**Figure S1**. *Lippia origanoides* Collected in the inflorescence period.


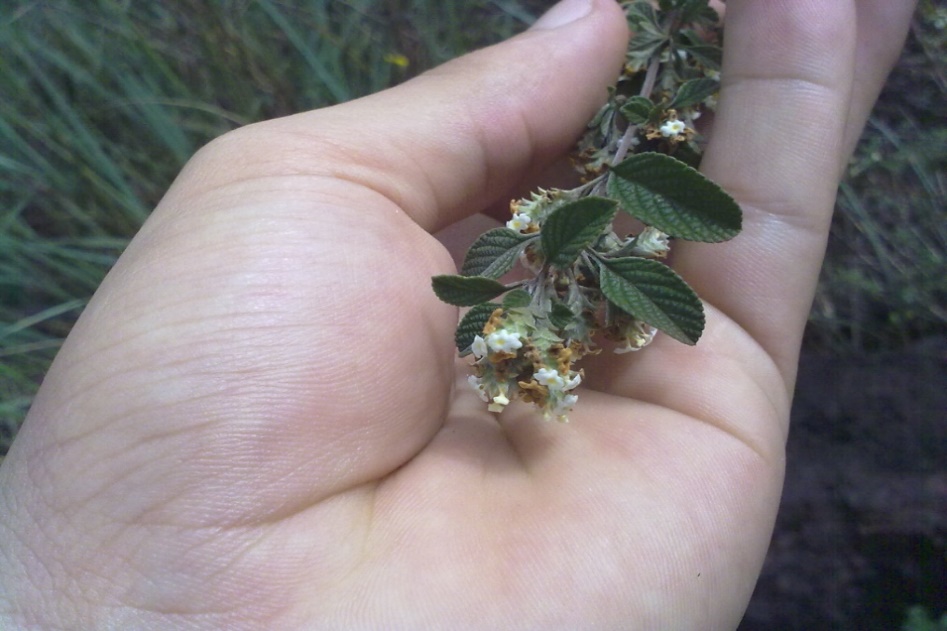


**Figure S2.** Modified Clevenger System for Hydrodestilation.

**
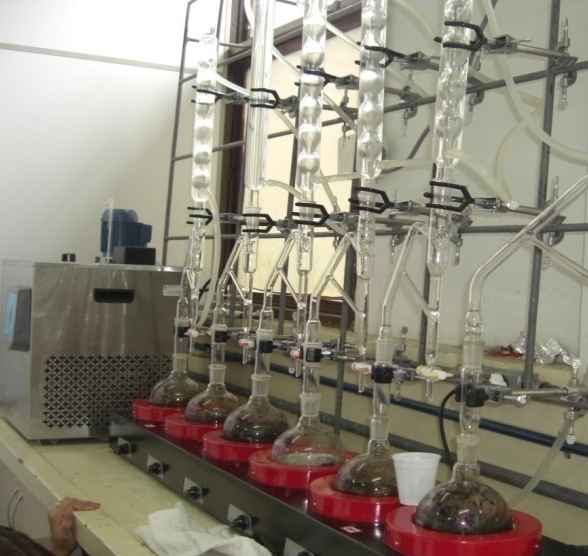
**

**Figure S3.** Obtaining the *L. Origanoids* essential oil.

**
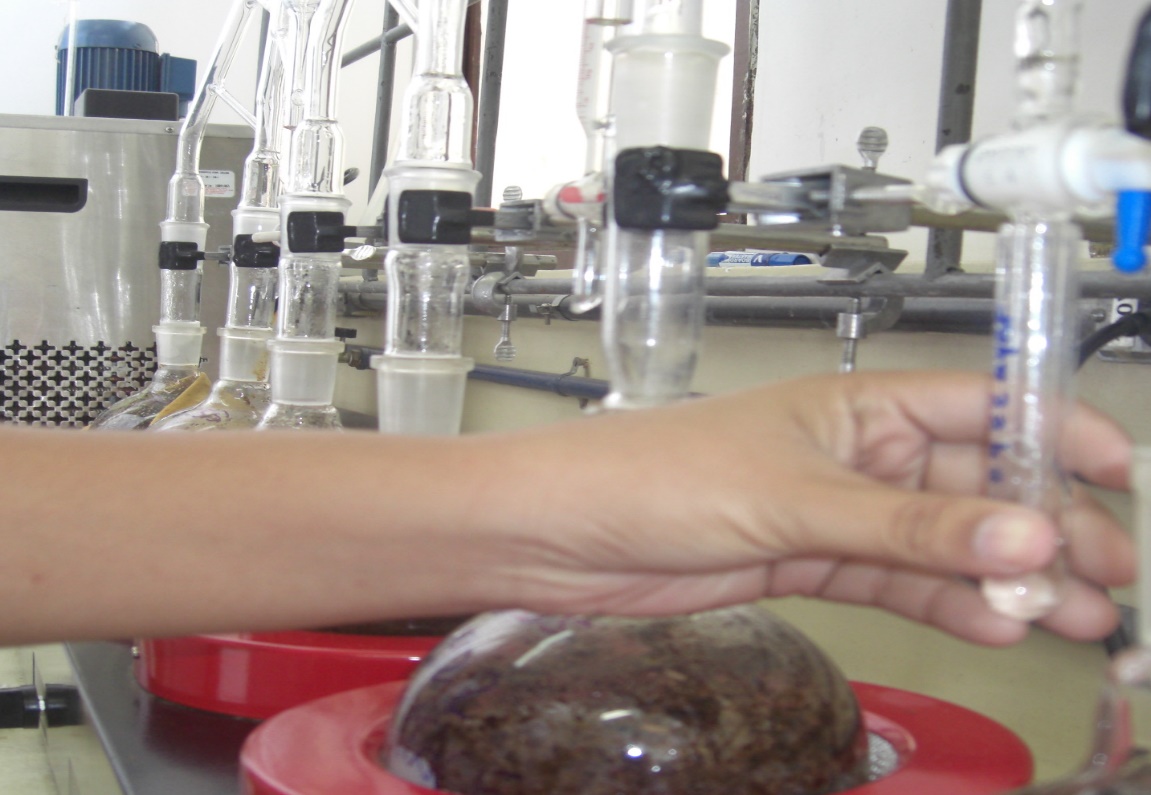
**

**Figure S4.** Agar BHI - Brain Heart Infusion


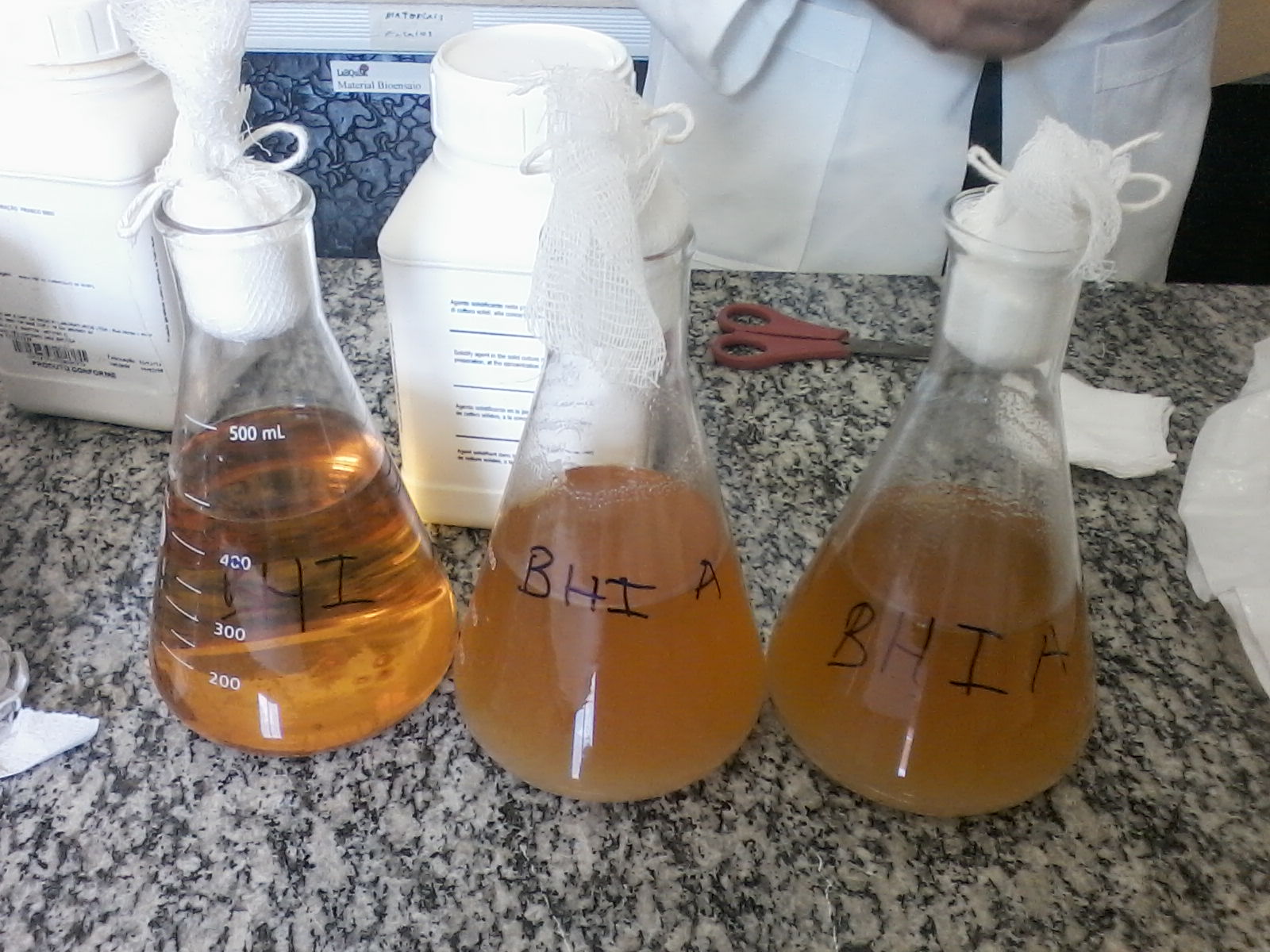


**Figure S5.** Minimum inhibitory concentration (CIM)


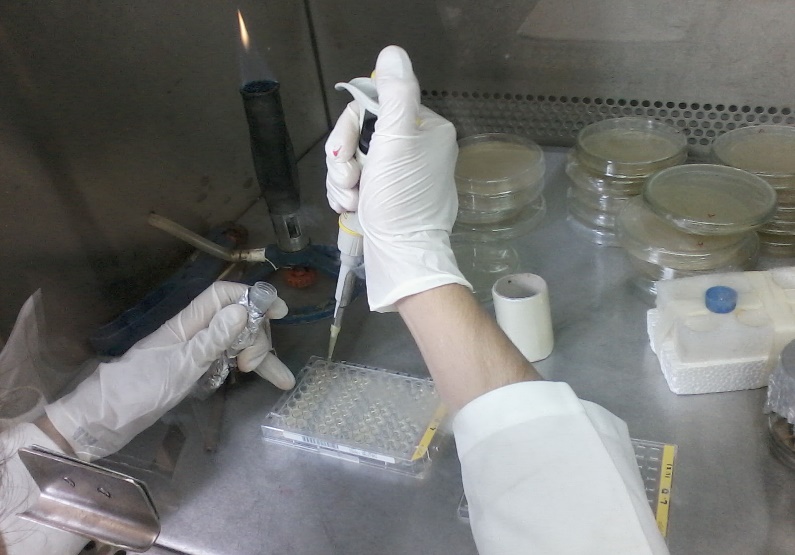


**Figure S6.** Minimum bactericidal concentration (CBM).


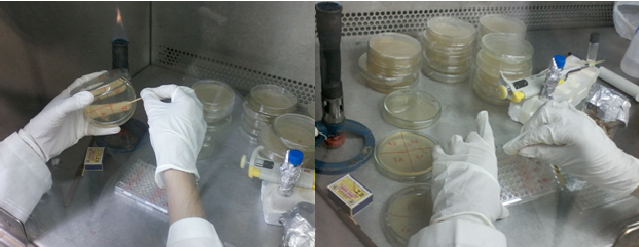

Supplement: Supplementary Materials — This section contains data regarding collection and experiments. [file 9930336.f1.docx]
